# Research on target localization and adaptive scrubbing of intelligent bathing assistance system

**DOI:** 10.3389/fbioe.2025.1550875

**Published:** 2025-05-09

**Authors:** Ping Li, Shikai Feng, Hongliu Yu

**Affiliations:** ^1^ Department of Biomedical Engineering, Changzhi Medical College, Changzhi, China; ^2^ Institute of Rehabilitation Engineering and Technology, University of Shanghai for Science and Technology, Shanghai, China

**Keywords:** intelligent bathing assistance, deep learning, multi-region skin detection, depth correction, hand-eye calibration, B-spline curve

## Abstract

**Introduction:**

Bathing is a primary daily activity. Existing bathing systems are limited by their lack of intelligence and adaptability, reliance on caregivers, and the complexity of their control algorithms. Although visual sensors are widely used in intelligent systems, current intelligent bathing systems do not effectively process depth information from these sensors.

**Methods:**

The scrubbing task of the intelligent bath assist system can be divided into a pre-contact localization phase and a post-contact adaptive scrubbing phase. YOLOv5s, known for its ease of deployment and high accuracy, is utilized for multi-region skin detection to identify different body parts. The depth correction algorithm is designed to improve the depth accuracy of RGB-D vision sensors. The 3D position and pose of the target point in the RGB camera coordinate system are modeled and then transformed to the robot base coordinate system by hand-eye calibration. The system localization accuracy is measured when the collaborative robot runs into contact with the target. The self-rotating end scrubber head has flexible bristles with an adjustable length of 10 mm. After the end is in contact with the target, the point cloud scrubbing trajectory is optimized using cubic B-spline interpolation. Normal vectors are estimated based on approximate triangular dissected dyadic relations. Segmented interpolation is proposed to achieve real-time planning and to address the potential effects of possible unexpected movements of the target. The position and pose updating strategy of the end scrubber head is established.

**Results:**

YOLOv5s enables real-time detection, tolerating variations in skin color, water vapor, occlusion, light, and scene. The localization error is relatively small, with a maximum value of 2.421 mm, a minimum value of 2.081 mm, and an average of 2.186 mm. Sampling the scrubbing curve every 2 mm along the x-axis and comparing actual to desired trajectories, the y-axis shows a maximum deviation of 2.23 mm, which still allows the scrubbing head to conform to the human skin surface.

**Discussion:**

The study does not focus on developing complex control algorithms but instead emphasizes improving the accuracy of depth data to enhance localization precision.

## 1 Introduction

The aging population trend places a substantial economic burden on families and insurance systems, creating an increased demand for specialized care, especially for bathing. Although various bathing aids are available, such as anti-slip mats, grab bars, and bath mats, most lack personalization, intelligent functions, and integrated functionality. The intelligent bathing assistance system offers a solution to caregiver shortages and the high demands on caregivers by minimizing awkward interactions and reducing potential risks for both users and caregivers. The system supports older adults’ independence and quality of life while advancing the intelligence and effectiveness of bathing assistance technologies (He et al., 2019).

With the advancement of artificial intelligence, visual perception has become one of the most prominent research areas, widely applied in fields such as drones (Shi et al., 2023), industrial robots (D'Avella et al., 2023), and service robots (Juang and Zhang, 2019). Lin et al. employed an RGB-D camera and utilized local feature descriptors of point clouds to achieve object recognition (Lin et al., 2019). Martínez et al. constructed composite feature vectors by integrating local and global features and employed feature fusion techniques to achieve clothing classification and perception based on an RGB-D camera (Martínez et al., 2019). Fu et al. developed a machine vision system based on an RGB-D camera, employing depth thresholding to remove background noise and utilizing a convolutional neural network to identify apples from RGB images (Fu et al., 2020). Luo et al. designed a vision perception system based on deep learning, capable of rapidly identifying wooden blocks within the field of view in industrial environments (Luo et al., 2020). Jia et al. employed a template-matching approach to automatically detect and segment cows from RGB and depth images (Jia et al., 2021). Yu et al. eliminated redundant information using depth images based on an RGB-D camera and trained a random forest binary classification model based on color and texture features to achieve lychee recognition (Yu L. et al., 2021). Huang et al. investigated the visual perception technology for assisting micro aerial vehicles in navigating stairs, employing a front camera to detect stairway entrances and a bottom camera to extract features of the stairs, walls, and railings (Huang and Lin, 2021). Weng et al. developed a dual-arm mobile robot visual perception system for human-robot interaction, using YOLO for target recognition and localization (Weng et al., 2022). Li et al. developed a tea-picking robot for field-based tea leaf recognition and localization based on an RGB-D camera, detecting tea bud regions with YOLO and employing point cloud data for 3D localization (Li Y. et al., 2021). Common methods for calculating the three-dimensional coordinates of targets often involve monocular vision, binocular vision, and RGB-D cameras, with typical data sources including RGB images, depth maps, and point clouds. The advantages and limitations of these data sources, as well as the sensors used, are summarized in Table 1. Bathing target localization requires three-dimensional information about the target, and visual sensors capable of generating depth information are the preferred choice for this task. Vision-based target localization offers advantages such as accessibility, universality, and the ability to provide rich scene information. However, in the context of bathing assistance, no template can represent all users, and manual labeling is not feasible. Moreover, the high safety requirements during scrubbing demand precise distance information. Additionally, variations in environmental factors, such as humidity and lighting conditions, pose challenges for skin detection.

**TABLE 1:** Advantages, limitations, and sensor types associated with various data sources.

Sensors	Data sources	Advantages	Limitations
Monocular vision	RGB images	Simple structure and low cost	Highly affected by lighting and unable to personally obtain depth information
Binocular vision	RGB images, depth images, and point cloud information	It can obtain deep information	Binocular matching is influenced by various factors, particularly its ineffectiveness in textureless scenes
RGB-D sensors	RGB images, depth images, and point cloud information	Capable of obtaining depth information with a variety of imaging principles available	Depth accuracy is closely related to the distance between the object and the sensor, with significantly reduced precision for transparent objects and reflective surfaces

Zlatintsi et al. developed the I-Support, which primarily consists of cameras, automatic scrubbers, and an electric shower chair (Zlatintsi et al., 2020). This system uses point cloud data for visual perception and employs predefined cleaning paths to simplify the problem. However, it uses multiple RGB-D cameras, leading to system redundancy, and involves complex control algorithms. Furthermore, it fails to meet personalized needs and cannot provide user-adaptive scrubbing capabilities.

Although previous studies have explored bathing assistance systems, they have not fully addressed the potential negative impact of depth value errors on bathing operations. To address this issue, this study designs a bathing assistance system and proposes a method to enhance the depth accuracy of visual sensors. Utilizing high-precision depth data, the study achieves accurate target localization and adaptive scrubbing functions. The structure of this paper is organized as follows: In the “Materials and Methods” section, the study provides a detailed discussion on target localization before contact and adaptive scrubbing after contact, with a focus on deep learning-based multi-region skin detection, depth value correction, and adaptive scrubbing. The “Results” section presents the outcomes of multi-region skin detection, localization experiments, and scrubbing experiments. The “Discussion” section offers an analysis of the experimental results. Finally, the “Conclusion” section summarizes the main findings and contributions of this research.

## 2 Materials and methods

**FIGURE 1:**
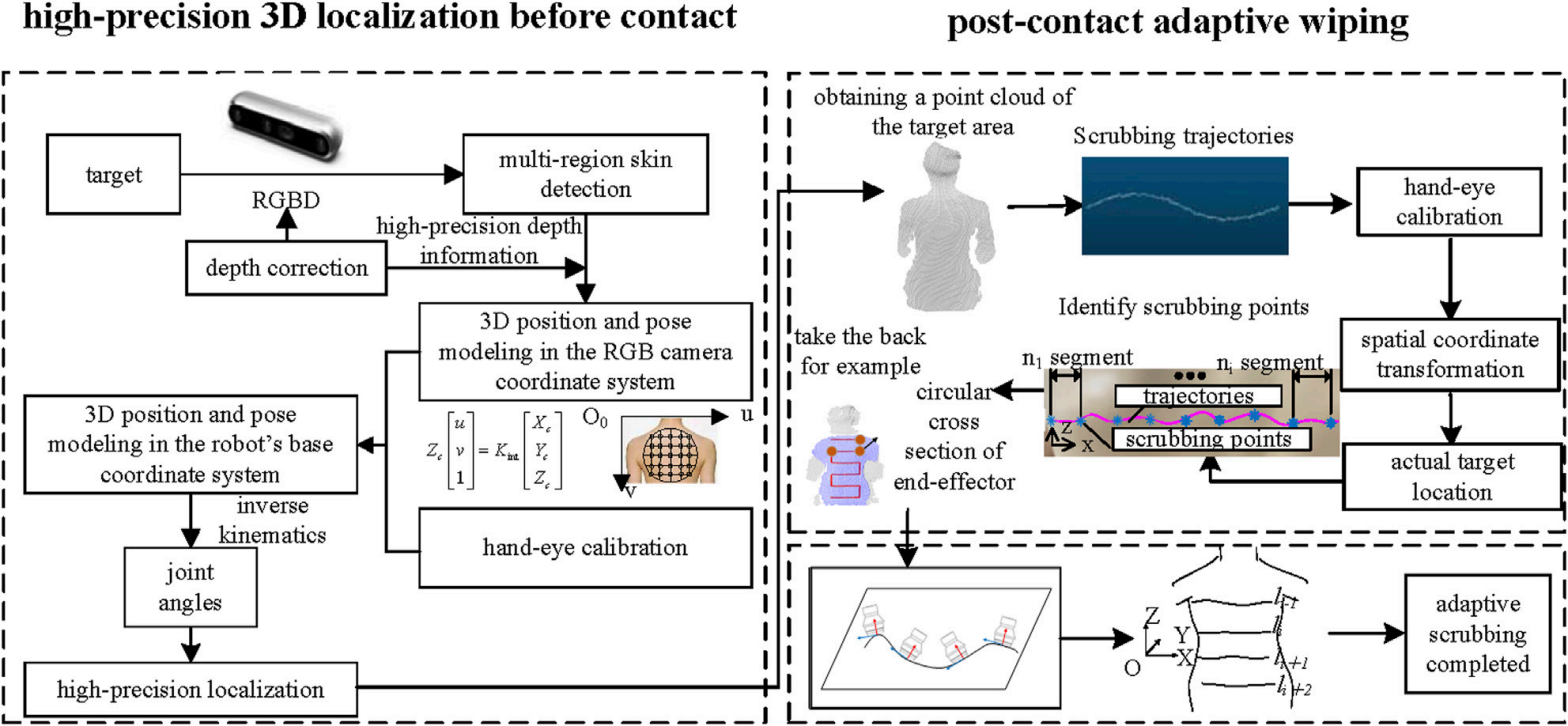
High-precision positioning before end-effector contact with the target, followed by adaptive scrubbing post-contact.

The bathing tasks of the intelligent bathing assistance system can be divided into two main phases: the pre-contact and post-contact tasks. The goal of the pre-contact phase is to achieve high-precision localization, ensuring that the end-effector accurately reaches the target area. The post-contact phase involves planning the adaptive scrubbing, ensuring that the end-effector conforms precisely to the surface of the human skin for scrubbing. As shown in Figure 1, after completing tasks such as skin detection, depth correction, 3D position and pose modeling, and hand-eye calibration, the system can execute the target localization task before contact. Once the end-effector reaches the target area, the system extracts and plans the scrubbing trajectory while updating the end-effector’s pose to perform adaptive scrubbing.

**FIGURE 2:**
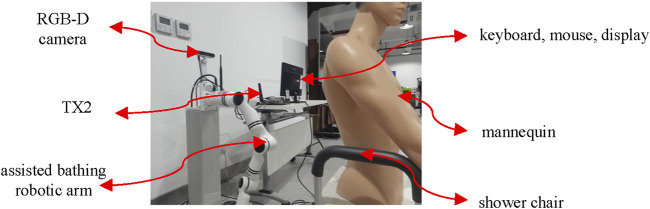
The experimental equipment.

The experimental equipment is shown in Figure 2. TX2 has a low price, low power consumption, and small size, making it suitable for situations such as cost control and limited workspace. The scheme of predicting depth by RGB increases the need for computational power and raises the cost. An RGB-D camera capable of generating depth information is preferred for this task. Due to the presence of water vapor and liquid droplets, the RGB-D camera based on the passive imaging principle is more suitable for applications in the assisted bathing scenario. The Intel RealSense D455 visual sensor demonstrates cost-effectiveness while maintaining superior imaging quality compared to its counterparts within the same product series. This device preserves identical field of view (FOV) specifications for both RGB and depth modalities, thereby enabling synchronized spatial registration that facilitates precise depth value extraction corresponding to individual RGB pixels. Considering the safety requirements and the special bathing environment, the bathing robotic arm needs to have force sensing and waterproof capabilities. Therefore, a lightweight six-degree-of-freedom collaborative robot arm RM65-6 F that meets the requirements is selected, with a six-degree-of-freedom force sensor installed at the end. The scrubbing head at the end-effector is capable of autonomous rotation and is fitted with flexible bristles.

### 2.1 Vision-based high-precision 3D localization before contact

#### 2.1.1 Deep learning-based multi-region skin detection

Skin detection plays a crucial role in diagnosing conditions such as melanoma and skin cancer (Khan et al., 2021a). In a robotic bathing system, the realization of different bathing modes necessitates the precise positioning of body parts to activate the appropriate mode for a specific body region. When using object detection for multi-region skin detection, the focus is on distinguishing between skin regions as opposed to classifying each pixel individually. This approach reduces both the annotation burden and computational complexity, thereby facilitating real-time robotic control (Li et al., 2023).

In recent years, object detection algorithms based on the deep convolutional neural network (DCNN) have advanced rapidly (Chen et al., 2024), utilizing large datasets to automatically learn features, demonstrating strong robustness to challenges such as steam, lighting, and target variations in bathing environments. DCNN-based object detection algorithms can be categorized into two-stage/single-stage approaches and anchor-based/anchor-free methods (Li P. et al., 2021). Among these, single-stage algorithms, which directly perform classification and regression, are particularly notable for their excellent real-time performance and have gained widespread attention in practical applications (Fang et al., 2022). The YOLOv5s model is distinguished by its minimalistic design, which is optimized for deployment on hardware platforms with restricted computational and memory footprints. It boasts an expedited inference rate, making it particularly well-suited for time-sensitive object detection scenarios (Lawal et al., 2023). YOLOv5s, with its ease of deployment (Yu Y. et al., 2021), real-time capabilities, and high accuracy, was selected as the preferred deep learning model for multi-region skin detection in this study.

##### 2.1.1.1 Construction of the datasets

**FIGURE 3:**
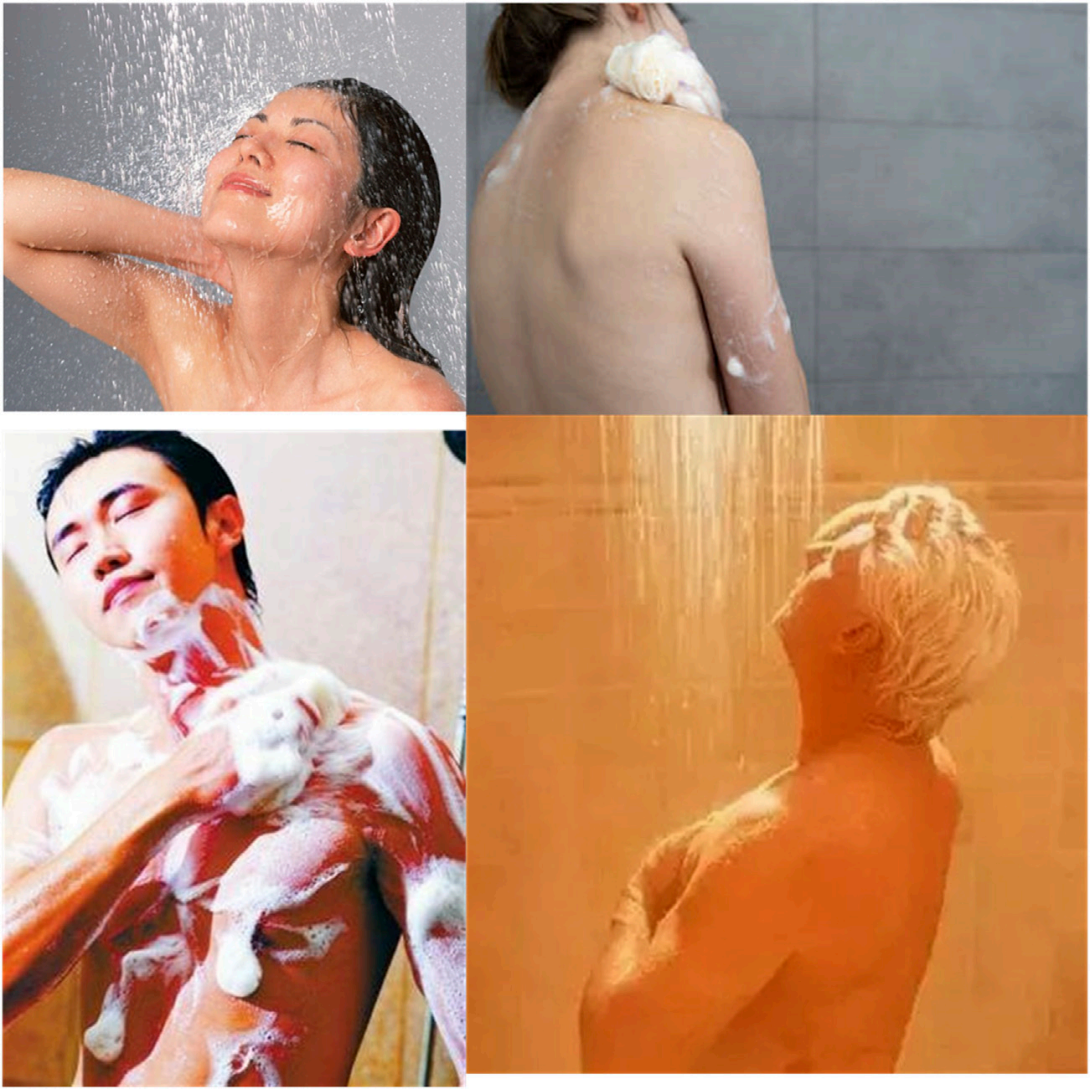
Example images of the object detection datasets.

To build diverse skin detection datasets, we collected images containing skin regions, considering factors such as skin tone, lighting conditions, and humidity. Using the annotation tool LabelImg (Geng et al., 2024; Hu et al., 2024), we manually delineated rectangular bounding boxes around each target and labeled their categories and positions, generating XML files in PASCAL VOC format. Each XML file includes the image filename, the coordinates of the ground truth (GT) bounding box, and the associated category labels. The datasets consisted of seven categories: (1) Face_skin, (2) Trunk_skin, (3) Upperlimb_skin, (4) Lowerlimb_skin, (5) Hand_skin, (6) Foot_skin, and (7) Background, representing the skin of various body parts and the background region. To address the issue of class imbalance and improve skin detection performance, we applied offline data augmentation techniques to ensure a more balanced distribution of samples across categories (Hussain et al., 2024). More than 20 methods were implemented, including adding random pixels, Gaussian noise, random rectangular occlusion, random pixel zeroing, salt-and-pepper noise, Gaussian blur, motion blur, adaptive histogram equalization, horizontal flipping, vertical flipping, proportional scaling, non-proportional scaling, random translation, HSV transformation, perspective transformation, random contrast adjustment, edge enhancement, random brightness adjustment, max pooling, average pooling, random cropping and padding, etc. These techniques were applied to images with limited sample sizes to generate new samples. In total, the datasets included 2,266 images, as shown in Figure 3.

##### 2.1.1.2 Model training settings and evaluation metrics

Training a network from scratch typically requires a substantial amount of annotated data (Pattnaik et al., 2020). However, collecting images containing skin is challenging, making it difficult to construct large-scale datasets. Training on small datasets poses a significant risk of model overfitting. To address this issue, we employed a transfer learning approach (Pratondo and Bramantoro, 2022), which enabled efficient training of a model on small datasets with performance comparable to training from scratch (Khan et al., 2021b; Mayya et al., 2024). Specifically, the model was pre-trained on the ImageNet dataset, and the resulting weight file served as the initial weight for the model.

The model was trained using the PyTorch framework at the Supercomputing Center of the University of Shanghai for Science and Technology. The datasets were split into training, validation, and test sets, with proportions of 60%, 20%, and 20%, respectively. The initial learning rate was set to 0.001, with a decay rate of 0.01, and the optimizer used was stochastic gradient descent (SGD). During training, the parameters of the backbone network were initially frozen, with only the remaining parameters updated. Afterward, the backbone network was unfrozen, and all parameters underwent trained. This strategy effectively enhanced the convergence speed and training efficiency of the network.

Recall and Precision are critical metrics for assessing the network performance, as shown in Equations 1, 2. Specifically, TP denotes the number of true positives (correctly detected targets), FP refers to the number of false positives, and FN represents the number of undetected targets (Lu and Hong, 2024). By plotting Recall on the x-axis and Precision on the y-axis, a Precision-Recall (P-R) curve can be generated. The area enclosed by the curve and the axes corresponds to the average precision (AP). The mean of the AP across all categories is defined as the mean average precision (mAP), which serves as an important evaluation metric for multi-class object detection.

$$Recall=\frac{TP}{TP+FN}$$ (1)

$$Precision=\frac{TP}{TP+FP}$$ (2)

#### 2.1.2 Depth correction algorithm

Upon localizing the region of the body part, acquiring distance measurements becomes imperative to facilitate accurate three-dimensional pose estimation. However, depth data from RGB-D cameras often exhibit issues such as high noise, low accuracy, and outliers (Alenya et al., 2014). Additionally, geometric distortions and system biases may affect these measurements (Yang et al., 2020), which can degrade localization accuracy. To enhance the precision of distance data, our study presents a depth correction algorithm to achieve high-accuracy target localization.

The depth quality evaluation metrics are essential for analyzing depth accuracy, developing depth correction algorithms, and evaluating the effectiveness of the corrections. *Z*_*accuracy*_ is used to assess the precision of depth data. The filling rate is represented by the ratio of valid pixels to total pixels. The root mean square error (RMSE) quantifies spatial noise. The filling rate, a hardware-dependent parameter that cannot be modified through algorithms, can be manually adjusted in Intel^®^ RealSense™ Viewer software. The *Z*_*accuracy*_ and RMSE are used as depth quality evaluation indicators in depth correction research. Their calculation methods are provided in Equations 3, and 4, where *i* denotes the index of a point in the point cloud, *n*_*0*_ represents the total number of points in the point cloud, *j*_*i*_ is the distance value at each point, GT denotes the ground truth distance, and *d*_*0i*_ is the distance from the *i*th point to the fitted plane. *Z*_*accuracy*_ quantifies the proximity between the measured depth values and the GT. It is calculated as the difference between the average depth value of all pixels and the GT. RMSE quantifies the intrinsic variation in depth values, calculated by measuring the deviation of all valid pixels from the best-fit plane (Gupta et al., 2023).

$$Z_{accuracy}=\frac{\sum_{i=1}^{n_{0}}\left|j_{i}-GT\right|}{n_{0}}$$ (3)

$$RMSE=\sqrt{\frac{\sum_{i=1}^{n_{0}}{d_{0i}}^{2}}{n_{0}}}$$ (4)

The D455 not only captures RGB and depth images of the scene but also outputs point cloud data simultaneously. Moreover, the point cloud and depth images can be converted interchangeably. The depth quality analysis of D455 reveals that the RMSE and *Z*_*accuracy*_ increase gradually with distance, exhibiting a nonlinear relationship. The depth measurement at each pixel deviates from the GT, while the average depth measurement across a planar surface also exhibits deviation from the GT. When capturing a flat wall, the error is reflected in two aspects: the deviation of individual 3D points from the true plane and the misalignment between the fitted plane and the actual plane. To correct the errors, each 3D point must be adjusted to align with the true plane, and the discrepancy between the fitted and actual planes requires rectification. A chessboard pattern is introduced for correction, leveraging its mature corner detection algorithms to facilitate accurate identification of the chessboard and its corresponding plane. As the error increases with distance, an iterative approach is employed. The correction begins at short distances, using parameters derived from these distances as initial values to estimate the error correction function for greater distances.

The depth correction algorithm is implemented in two stages: (1) The function *f*_*l*_ is utilized to correct depth errors at different pixels. A specific function *f*_*l*_ should be calculated for each pixel to correct the 3D points of a flat wall surface, ensuring they align onto a single plane. (2) The function *f*_*g*_ is employed to correct the average depth value, ensuring the plane from the previous stage is aligned to its true position. In theory, the chessboard pattern should form a planar surface (Fryskowska, 2019). The chessboard plane observed within the RGB camera’s field of view is aligned with the corrected point cloud plane from the first stage to estimate the errors. Since the RGB image and the point cloud are in different reference frames, there exists a rigid-body transformation between the two. To perform function fitting, the transformation matrix ^***C***^_***D***_ ***M*** between the RGB image and the point cloud must be obtained. Additionally, the process of calculating the extrinsic parameters relies on the accuracy of the intrinsic parameters (Zhang et al., 2020). To address the issue of interdependence, a solution that simultaneously optimizes both *f*_*g*_ and ^***C***^_***D***_ ***M*** is adopted.

**FIGURE 4:**
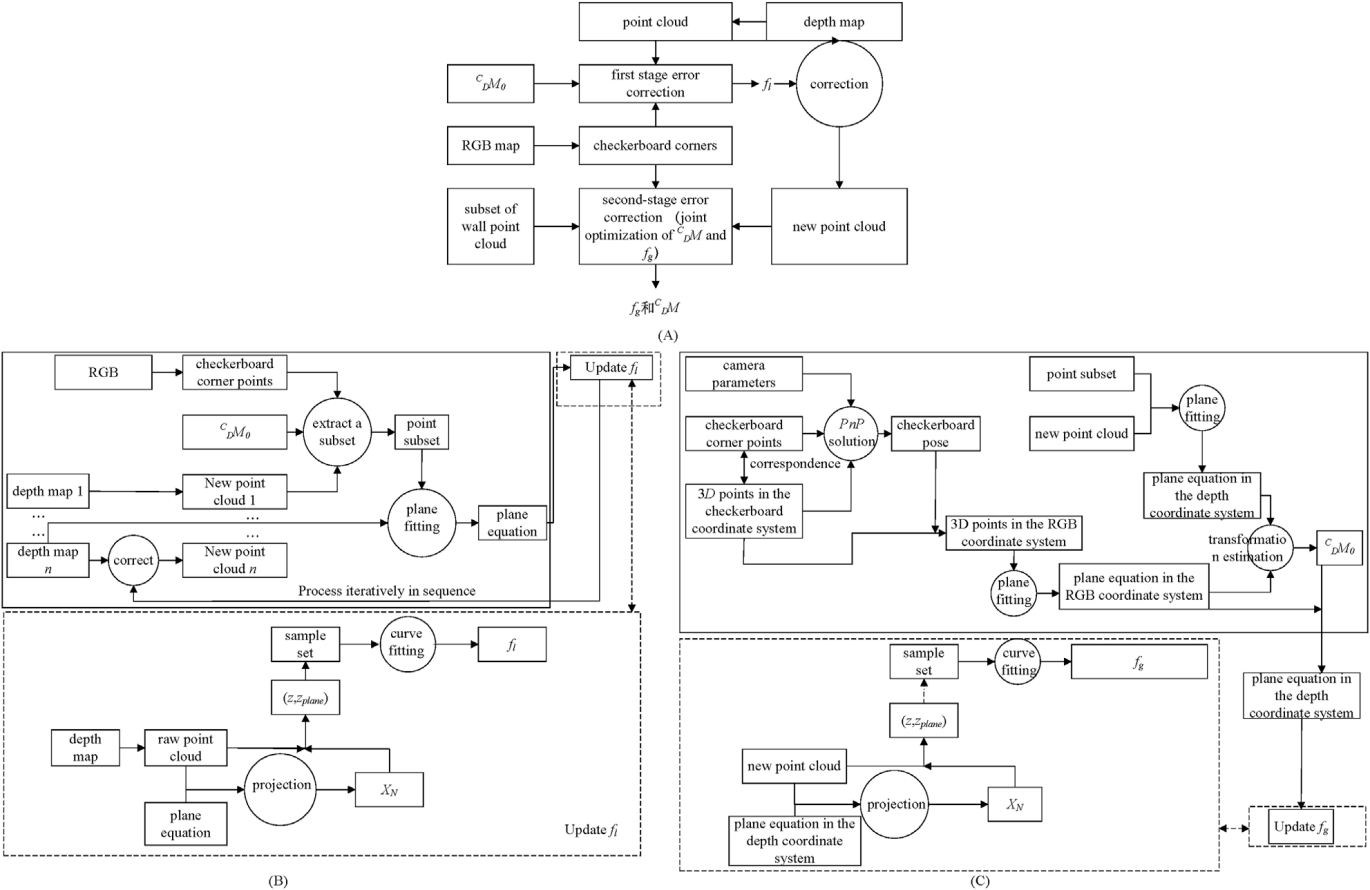
Depth correction **(A)** Two-stage depth correction algorithm **(B)** *f*_*l*_ estimation methods **(C)** *f*_*g*_ estimation methods.

The depth correction algorithm is illustrated in Figure 4A. The chessboard corner points are first extracted from the RGB image, and a point cloud is generated from the depth image. Function *f*_*l*_ is estimated using the chessboard corner points, the corresponding point cloud, and the factory-calibrated transformation matrix ^*C*^_*D*_ *M*_*0*_. Once *f*_*l*_ is determined, it is applied to correct the point cloud. The corrected point cloud, chessboard corner points, and a subset of the wall point cloud data are then combined to calculate function *f*_*g*_ and ^***C***^_***D***_ ***M***.

The process of estimating *f*_*l*_ is illustrated in Figure 4B. A chessboard is pasted on a flat wall, and the camera position is progressively adjusted to increase the distance from the wall. Each collected point cloud is processed iteratively. The current estimate of *f*_*l*_ is applied to the initial point cloud to correct the errors. Wall points are extracted from the corrected point cloud, and a plane is fitted to the subset to improve computational efficiency (Zhu et al., 2021). The plane is then used to update *f*_*l*_. Data for fitting *f*_*l*_ are extracted through distance-based projection method, with each processed corrected point cloud generating a set of data for fitting *f*_*l*_. The iterative process continues for all point clouds, resulting in the progressive refinement of *f*_*l*_.

The process of estimating *f*_*g*_ is illustrated in Figure 4C. In the same scene, the plane in the RGB image and the corresponding point cloud can be aligned through specific rotational and translational transformations. The rotation matrix and translation vector represent the rigid-body transformation between the RGB camera coordinate system and the depth camera coordinate system (Yang et al., 2020). The transformation is estimated using the plane equations derived from the RGB image and the corresponding point cloud. This transformation is subsequently applied to the plane equation in the RGB camera coordinate system to derive the plane equation in the depth camera coordinate system, thereby updating *f*_*g*_.

The D455 camera was used to collect data from a flat wall equipped with a chessboard pattern at varying distances and orientations. RGB images, depth images, and point clouds were captured for estimating *f*_*l*_ and *f*_*g*_.

#### 2.1.3 3D position and pose modeling of target points in the RGB camera coordinate system

According to the camera model, if the distance *Z*_*c*_ of the target point and the camera intrinsic matrix ***K***_***int***_ are known, the 3D coordinates of the target point in the RGB camera coordinate system can be derived from its pixel coordinates [u,v] in the RGB image, as shown in Equation 5 (Konecny et al., 2024). This enables 3D positional modeling of the target point in the RGB camera coordinate system, denoted as [*X*_*c*_,*Y*_*c*_,*Z*_*c*_].

$$Z_{c}\left[\begin{array}{c} u\\ v\\ 1 \end{array}\right]=K_{\mathrm{int}}\left[\begin{array}{c} X_{c}\\ Y_{c}\\ Z_{c} \end{array}\right]$$ (5)

**FIGURE 5:**
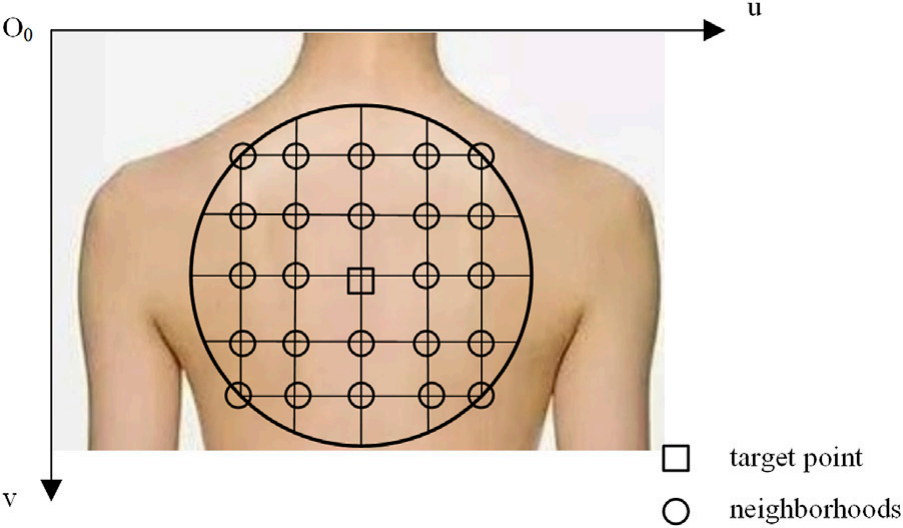
Attitude modeling at the target point.

As shown in Figure 5, *O*_*0*_-*uv* represents the pixel coordinate system in the camera model. A circle is drawn with the target point as the center and a predefined radius. By utilizing the pixel coordinates and corresponding depth values, the 3D positions of all pixels within the circle in the RGB camera coordinate system are computed. These points define a surface on the target surface, and the pose of the target point can be modeled through surface normal vector analysis. The issue of visual occlusion can be addressed by adjusting the circle’s radius.

#### 2.1.4 Hand-eye calibration

The camera-robot calibration paradigm is conventionally categorized into Eye-in-Hand and Eye-to-hand configurations. In the Eye-in-hand system, the sensor is mounted on the robotic end-effector, enabling complete observation of the target workspace and facilitating precise visual servo control. This configuration, however, presents inherent challenges including motion-induced image degradation and compounded error propagation from kinematic inaccuracies during calibration procedures. Conversely, the Eye-to-hand system positions the camera in a stationary configuration external to the robotic manipulator, thereby eliminating motion artifacts while achieving extended spatial coverage encompassing both the target domain and manipulator workspace. This arrangement introduces potential visual occlusion risks.

Considering operational reliability and environmental factors such as humidity resistance requirements, the solution adopted an Eye-to-hand configuration. During hand-eye calibration, the D455 was mounted on the robot’s base, and the calibration was performed using the ROS package *easy_handeye* and an ArUco board attached to the robot’s end-effector. The ArUco board was generated by the ArUco markers generator with Marker ID 582 and Marker size 100 mm. The process provided the translation vector components *t*_*x*_、*t*_*y*_, and *t*_*z*_, along with the quaternion components *x*、*y*、*z*, and *w* representing the orientation. The 3D position and pose of the target point were then transformed from the RGB camera coordinate system to the robot’s base coordinate system (Ding et al., 2024). Subsequently, inverse kinematics was applied to compute the joint angles, enabling precise positioning and contact with the skin.

### 2.2 Post-contact adaptive wiping

When the end effector establishes contact with the target skin area, we must plan a wiping trajectory to accommodate the curvature of the human skin.

#### 2.2.1 Point cloud-based optimization of scrubbing trajectories and normal vector calculation

Cubic B-spline interpolation is utilized to optimize the point cloud trajectory, ensuring a smooth wiping path. B-spline curves have been extensively applied in trajectory fitting and discretization. For a given degree k, a k-degree B-spline curve *p*(*μ*) is defined by Equation 6, where *U* = {*μ*_*0*_, *μ*_*1*_, … , *μ*_*k*_, *μ*_*k+1*_, … , *μ*_*m-k-1*_, *μ*_*m-k*_, … , *μ*_*m*_} represents the knot vector, and *N*_*j,k*_(*μ*) denotes the j-th k-degree B-spline basis function, as expressed in Equation 7 (Guo et al., 2024). Using the deBoor algorithm (Kong et al., 2016; Wang et al., 2019), we derive Equation 8. The arc length is approximated through step size, yielding Equation 9 where ∆*μ*_*i*_^*P*^ represents the parameter increment for the *i*-th interpolation step (*i* = 1, 2, 3, … , *n*), and *l* denotes the interpolation step length. The *μ*-value corresponding to the *i*-th interpolation point can be calculated through Equation 10.

$$p\left(\mu \right)=\sum_{j=i-k}^{i}d_{j}N_{j,k}\left(\mu \right),\mu \in \left[\mu_{i},\mu_{i+1}\right],i=k,k+1,\cdots n$$ (6)

$$\left\{\begin{array}{l} N_{i,0}\left(\mu \right)=\left\{\begin{array}{l} 1,\mu_{i}\leq \mu \leq \mu_{i+1}\\ 0,else \end{array}\right.\\ {N_{i,k}\left(\mu \right)=\frac{\mu -\mu_{i}}{\mu_{i+k}-\mu_{i}}N}_{i,k-1}\left(\mu \right)+{\frac{\mu_{i+k+1}-\mu }{\mu_{i+k+1}-\mu_{i+1}}N}_{i+1,k-1}\left(\mu \right)\\ set\frac{0}{0}=0 \end{array}\right.$$ (7)

$$\frac{d}{d\mu }p\left(\mu \right)=\sum_{j=i-k}^{i}d_{j}\frac{d}{d\mu }N_{j,k}\left(\mu \right)=\sum_{j=i-k}^{i}d_{j}\frac{N_{i,k-1}\left(\mu \right)}{\mu_{i+k}-\mu_{i}}-\frac{N_{i+1,k-1}\left(\mu \right)}{\mu_{i+k+1}-\mu_{i+1}},\mu \in \left[\mu_{i},\mu_{i+1}\right]$$ (8)

$$∆\mu_{i}^{P}=\frac{l}{\sqrt{{\left(\frac{d}{d\mu }x_{i}\left(\mu \right)\right)}^{2}+{\left(\frac{d}{d\mu }y_{i}\left(\mu \right)\right)}^{2}+{\left(\frac{d}{d\mu }z_{i}\left(\mu \right)\right)}^{2}}}$$ (9)

$$\mu_{i}^{P}=\sum_{i=1}^{i}∆\mu_{i}^{P}$$ (10)

**FIGURE 6:**
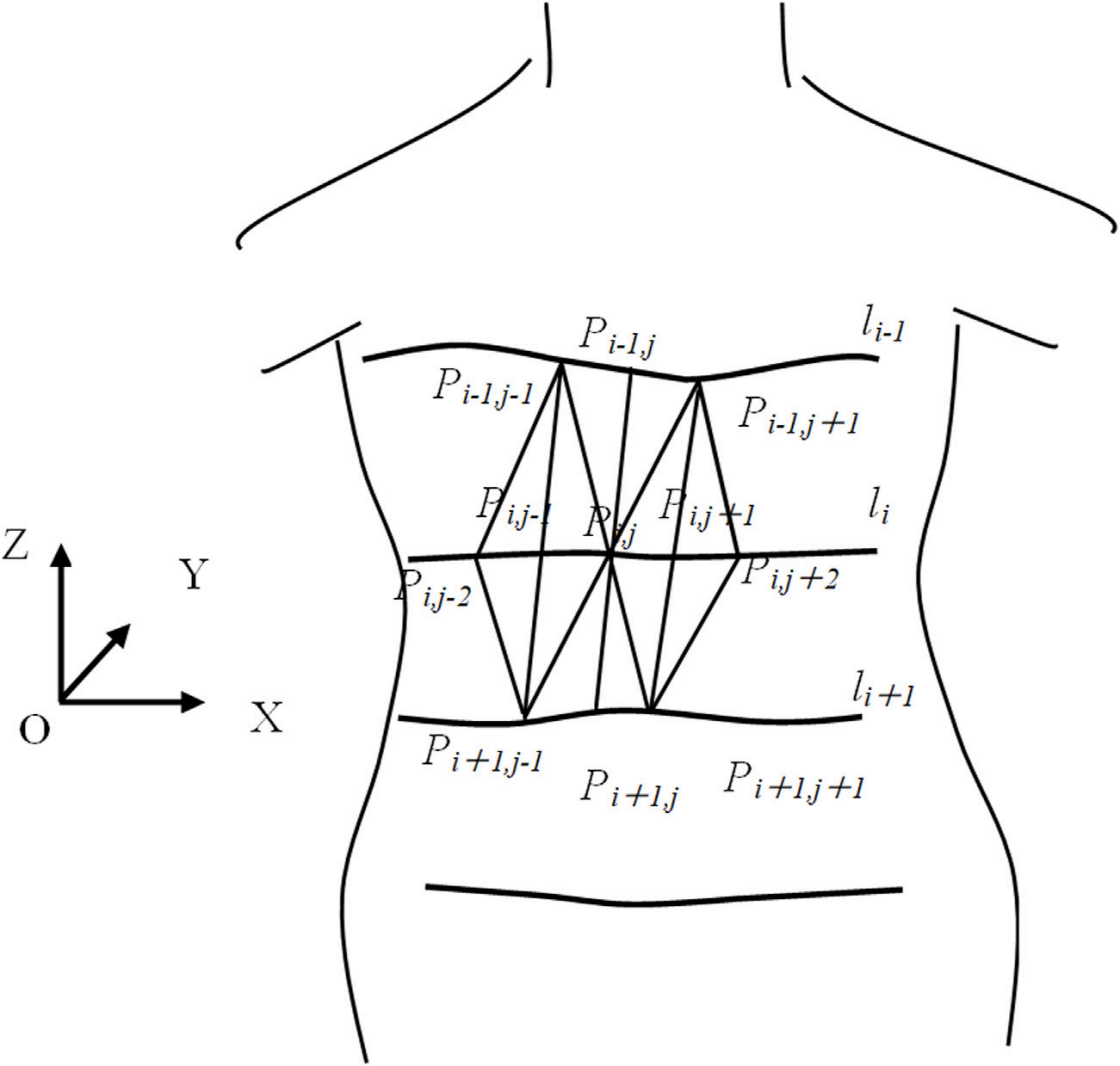
Topological relationships between points.

To establish topological relationships between points in the cloud, we employ approximate triangular tessellation to generate a triangular mesh, as illustrated in Figure 6. Let {*l*_*1*_,*l*_*2*_, … ,*l*_*n*_} represent the set of wiping paths, where *P*_*i,j*_ denotes a wiping point. *P*_*i,j-1*_ and *P*_*i,j+1*_, located on the same scan line as *P*_*i,j*_, and *P*_*i-1,j*_ and *P*_*i+1,j*_, which are the closest points to *P*_*i,j*_ on adjacent scan lines, are identified. Using these points, four triangles are constructed with the following vertices: {*P*_*i,j*_,*P*_*i,j+1*_,*P*_*i-1,j*_}, {*P*_*i,j*_,*P*_*i-1,j*_,*P*_*i,j-1*_}, and {*P*_*i,j*_,*P*_*i+1,j*_,*P*_*i,j+1*_}. The coordinates of the triangle vertices are denoted as (*P*_*x1*_, *P*_*y1*_, *P*_*z1*_), (*P*_*x2*_, *P*_*y2*_, *P*_*z2*_), and (*P*_*x3*_, *P*_*y3*_, *P*_*z3*_), and the method for calculating the normal vector is given by Equations 11, 12, 13. For the shared vertex *P*_*i,j*_, the normal vector *n*_*i,j*_ is approximated as the weighted average of the normal vectors of the four triangles, with the area of each triangle serving as the weight. For boundary points, the normal vector is calculated by taking the weighted average of the normal vectors of the two adjacent triangles that share the vertex.

$$\left\{\begin{array}{c} \overset{´}{n_{x}}=\left(P_{y2}-P_{y1}\right)\left(P_{z3}-P_{z2}\right)-\left(P_{z2}-P_{z1}\right)\left(P_{y3}-P_{y2}\right)\\ \overset{´}{n_{y}}=\left(P_{z2}-P_{z1}\right)\left(P_{x3}-P_{x2}\right)-\left(P_{x2}-P_{x1}\right)\left(P_{z3}-P_{z2}\right)\\ \overset{´}{n_{z}}=\left(P_{x2}-P_{x1}\right)\left(P_{y3}-P_{y2}\right)-\left(P_{y2}-P_{y1}\right)\left(P_{x3}-P_{x2}\right) \end{array}\right.$$ (11)

$$\delta =\sqrt{{\overset{´}{n_{x}}}^{2}+{\overset{´}{n_{y}}}^{2}+{\overset{´}{n_{z}}}^{2}}$$ (12)

$$\left\{\begin{array}{c} n_{x}=\frac{\overset{´}{n_{x}}}{\delta }\\ n_{y}=\frac{\overset{´}{n_{y}}}{\delta }\\ n_{z}=\frac{\overset{´}{n_{z}}}{\delta } \end{array}\right.$$ (13)

#### 2.2.2 Real-time trajectory planning

**FIGURE 7:**
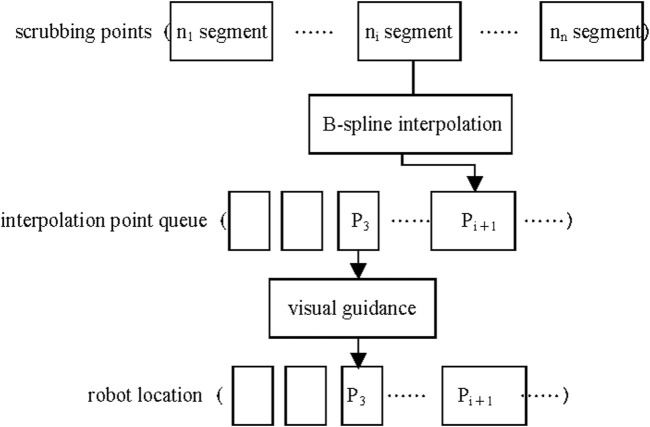
First-in-first-out queue for interpolated points.

When the wiping path becomes lengthy, the increasing number of interpolation and fitting points elevates computational load, thereby degrading real-time performance. Furthermore, unexpected human movements during operation may alter the wiping path. To resolve these issues, we propose a segmented interpolation real-time planning method as illustrated in Figure 7. This method divides the wiping trajectory into several segments and performs interpolation for each segment sequentially. The interpolation points are stored in a First-In-First-Out (FIFO) queue, where the control function retrieves points to guide the robot’s motion. With this approach, the robot can initiate movement after first-segment interpolation. Both B-spline interpolation and motion guidance processes are executed in separate threads for concurrent execution. This method not only facilitates real-time trajectory planning but also enhances adaptability to unexpected movements. If unforeseen movements occur during the robot’s wiping process, real-time adjustments can be made during the fitting of the next trajectory segment. Increased segmentation granularity enhances robustness against unexpected human motions.

#### 2.2.3 Updating the pose of the scrubbing head

**FIGURE 8:**
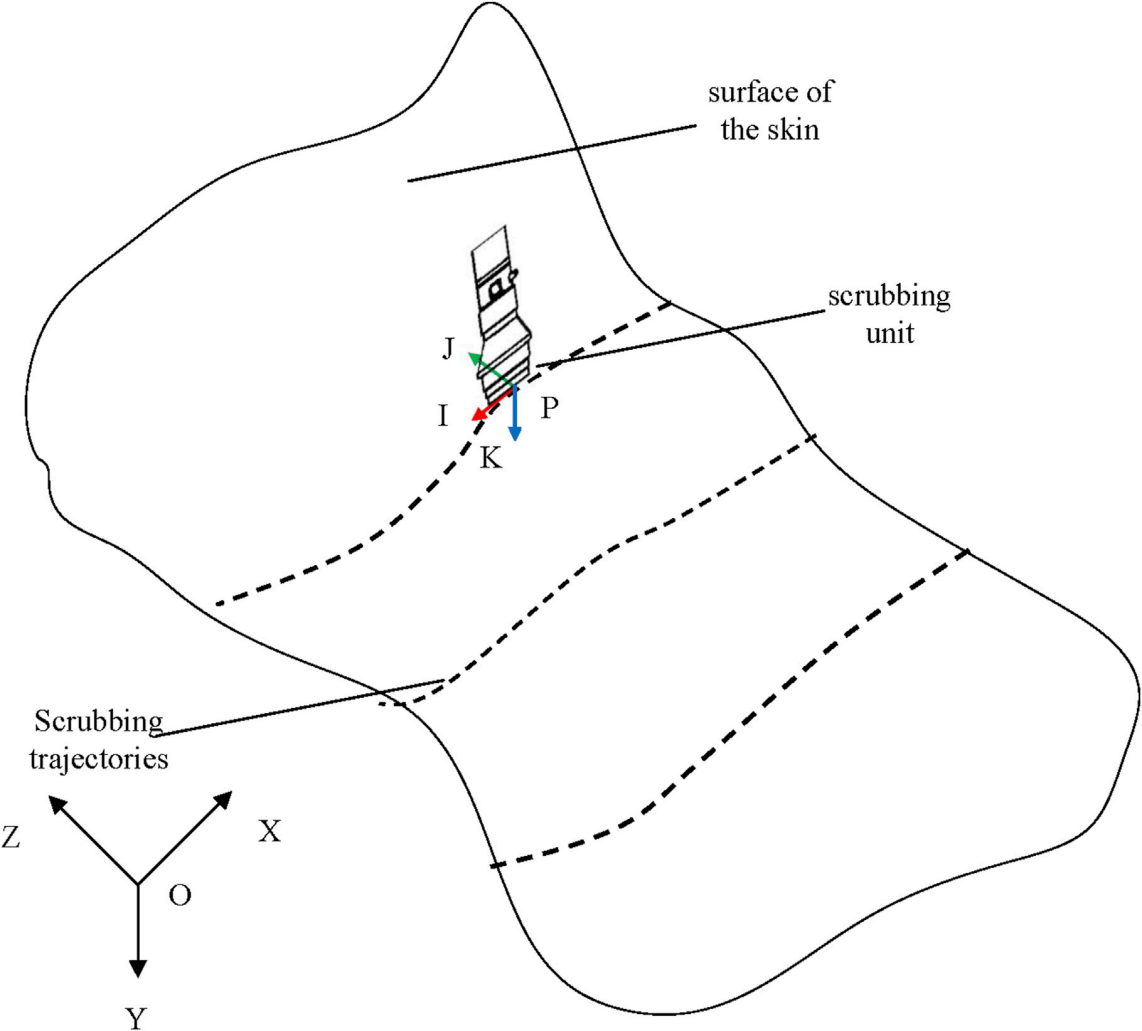
Scrubbing schematic.

The pose of the wiping head must be updated in real-time according to the dynamic changes in the wiping trajectory, which ensures proper contact with the target surface, thereby improving wiping efficiency and maintaining stable contact. As shown in Figure 8, the pose at the scrubbing point P is [*p*_*x*_, *p*_*y*_, *p*_*z*_, *φ*_*x*_, *φ*_*y*_, *φ*_*z*_], and the scrubbing head vector is K = [-*a*_*x*_, -*a*_*y*_, -*a*_*z*_]. Let the positive direction of the I-axis represent the tangential direction at P, with the tangential vector defined as *I* = [*o*_*x*_, *o*_*y*_, *o*_*z*_]. The J-axis is the cross product of K and I, expressed as *J* = [*m*_*x*_, *m*_*y*_, *m*_*z*_]. The transformation matrix ^*B*^ *P*_*H*_ from the tool coordinate frame (defined by I, J, K) to the robot base coordinate frame is given by Equation 14. The scrubbing pose is transformed into the end-effector pose of the robot, as shown in Equation 15. Here, ^*B*^_*E*_ *T*_*P*_ is the pose matrix of the robot during scrubbing, ^*H*^_*P*_ *T* denotes the transformation matrix from the scrubbing tool coordinate frame to the local coordinate frame of the scrubbing point, and ^*E*^_*H*_ *T* is the transformation matrix from the tool coordinate frame to the end flange coordinate frame. During scrubbing, the tool coordinate frame coincides with the local coordinate frame of the scrubbing point, i.e., ^*H*^_*P*_ *T* = *I*, which is the identity matrix. The length of the scrubbing head d = 10 cm; thus, ^*E*^_*H*_ *T* is defined as shown in Equation 16. Consequently, the robot’s pose matrix is expressed in Equation 17. By integrating the inverse kinematics model, the joint angles for the robot are calculated.

PHB=mxoxmyoyaxpxaypymzoz00azpz01 (14)

TPEBTHETPH=PHB (15)

THE=1001000000001d01 (16)

TPEB=PHBTHE−1 (17)

The angles *θ*_*i*_, formed by adjacent vectors ***P***_***i-1***_ ***P***_***i***_ and ***P***_***i***_ ***P***_***i+1***_ within the trajectory point sets {*P*_*i,1*_,*P*_*i,2*_, … , *P*_*i,n*_}, are calculated. These angles represent the rotation angle of the scrubbing head in the base coordinate system, denoted as *θ*_*z*_ = *θ*_*i*_. The calculation method for *θ*_*i*_ is shown in Equation 18. To reduce computational complexity, a threshold angle *θ* = 6°is set: if *θ*_*z*_<*θ*, no adjustment is made to the scrubbing head's pose. The threshold value is determined through extensive scrubbing experiments. When *θ*_*i*_ is below 6°, indicating minimal contour variation, no pose adjustment is required. This approach reduces computation while ensuring task completion.

$$\theta_{i}=\cos^{-1}\left(\frac{P_{i-1}P_{i}\cdot P_{i}P_{i+1}}{\left|P_{i-1}P_{i}\right|\cdot \left|P_{i}P_{i+1}\right|}\right)\left(i=1,2,\cdots ,n-1\right)$$ (18)

## 3 Results

### 3.1 Multi-region skin detection

Upon completion of training, YOLOv5s had a compact model size of 27 MB, yet achieved a mAP of 90%. When deployed on the TX2 platform, YOLOv5s demonstrated a frame rate of 42 frames per second (FPS), enabling real-time detection performance.

**FIGURE 9:**
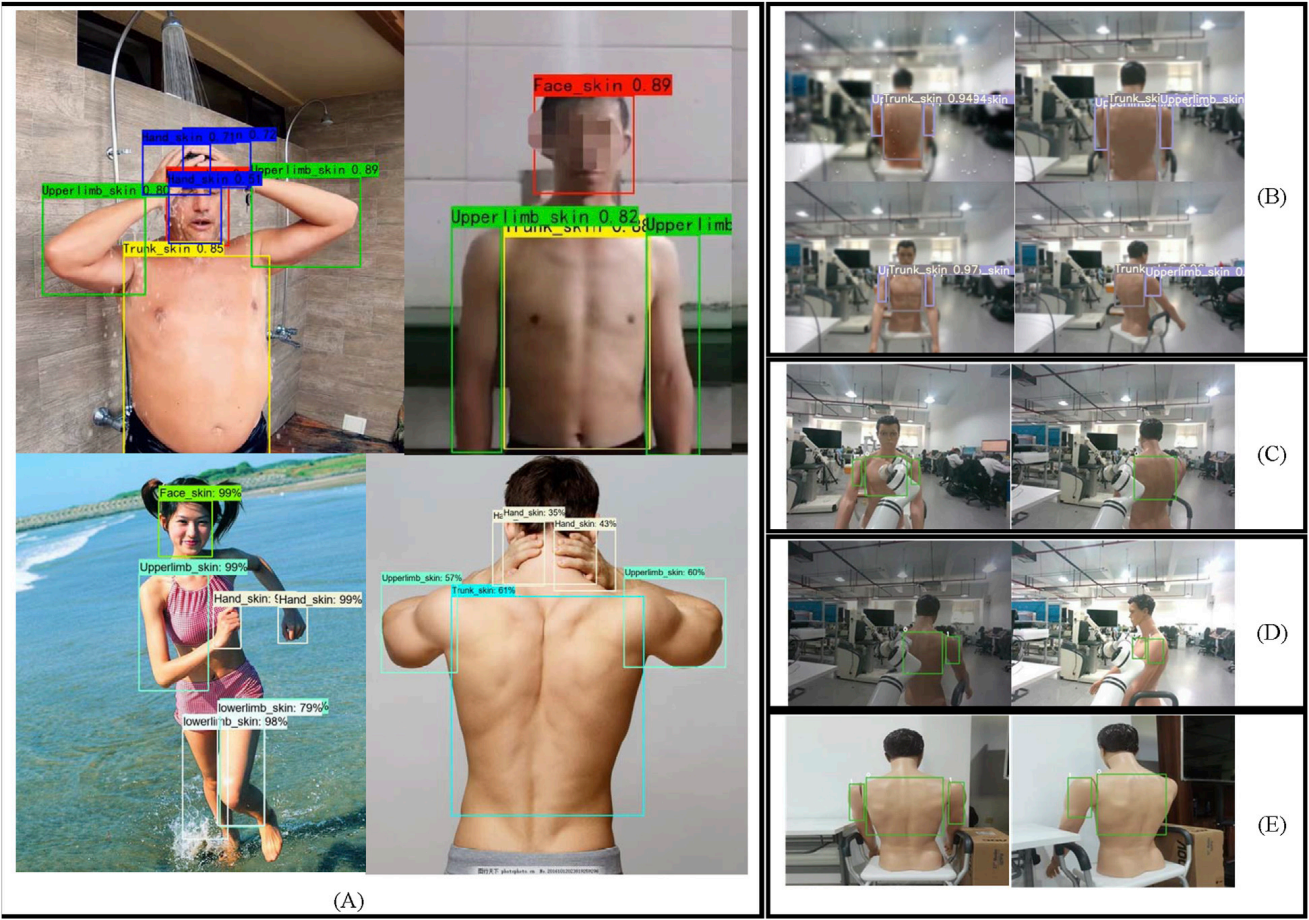
Robustness testing of the model **(A)** Detection results across different skin tones **(B)** Detection results in different humidity scenarios **(C)** Inference results under occlusion **(D)** Inference results under different lighting conditions **(E)** Inference results across different scenarios.

The robustness test results are shown in Figure 9. YOLOv5s demonstrates a certain level of robustness to variations in skin tone, humidity, lighting, occlusion, and scene conditions. As shown in panel (A), the model accurately detects skin across fair, medium, and dark tones. Panel (B) illustrates the application of image processing techniques on bathing scene images containing human models, simulating the humid environment commonly encountered during bathing. In such conditions, YOLOv5s still accurately detects the skin across various body regions. Panels (C) (D), and (E) further demonstrate the model’s robustness to occlusion, lighting variations, and different scene contexts.

### 3.2 Localization experiments

Considering that the center of the detection box was most likely to be located within the skin region, the center point was selected as the target point. The actual position in the base coordinate system was determined using skin detection, depth information, camera intrinsic parameters, and the hand-eye matrix. The ideal position was defined as the location data displayed on the teach pendant when the six-dimensional force sensor was adjusted to zero. The human model’s position was adjusted, and eight localization tests were conducted. Localization errors were computed based on Equation 19, where the subscript *l* represents the theoretical position and *s* represents the actual position. The error along each of the three axes was taken as the absolute difference between the actual and theoretical positions. The impact of the depth correction algorithm on localization performance was evaluated by comparing the differences between the actual and ideal depth data. The actual data was obtained from the teach pendant reading after the robot’s localization, while the ideal data corresponded to the teach pendant reading when the six-dimensional force sensor was set to zero. The error was calculated as the absolute difference between these two readings.

$$\varepsilon =\sqrt{{\left(x_{l}-x_{s}\right)}^{2}+{\left(y_{l}-y_{s}\right)}^{2}+{\left(z_{l}-z_{s}\right)}^{2}}$$ (19)

The results were presented in Table 2. The robotic arm’s localization error was relatively small, with a maximum value of 2.421 mm, a minimum value of 2.081 mm, and an average of 2.186 mm. The average errors along the x, y, and z-axes were 1.144 mm, 1.368 mm, and 1.111 mm, respectively. Before and after depth correction, the mean errors were 18.329 mm and 1.111 mm, respectively. These results indicated that the localization error and the errors along the three axes were minimal, demonstrating high localization accuracy. Moreover, depth correction was critical for achieving high-precision localization.

**TABLE 2:** Results of the positioning experiment.

No.	Theoretical position (mm)	Actual position (mm)	Positioning error (mm)	*X*-axis error (mm)	*Y*-axis error (mm)	*Z*-axis error (mm)	Pre-correction depth error (mm)
1	(251.04, 375.06, 763.69)	(252.15, 376.14, 765.15)	2.128	1.11	1.08	1.46	21.02
2	(292.02, 272.92, 833.79)	(292.91, 271.60, 832.45)	2.081	0.89	1.32	1.34	19.35
3	(242.42, 162.88, 725.62)	(241.38, 164.74, 725.55)	2.132	1.04	1.86	0.07	14.87
4	(246.15, 344.01, 631.70)	(247.87, 345.25, 632.40)	2.233	1.72	1.24	0.7	14.45
5	(246.56, 344.50, 631.16)	(247.62, 343.40, 632.66)	2.141	1.06	1.1	1.5	16.34
6	(328.02, 148.08, 855.10)	(329.41, 149.25, 856.70)	2.421	1.39	1.17	1.6	24.34
7	(253.3, 373.52, 762.91)	(254.10, 375.28, 764.01)	2.224	0.8	1.76	1.1	17.28
8	(327.11, 147.31, 854.44)	(328.25, 148.72, 853.32)	2.131	1.14	1.41	1.12	18.98

The proposed depth correction methodology utilized a multi-modal dataset comprising synchronized RGB images, depth maps, and 3D point clouds. A comparative analysis was conducted against the framework presented in the literature (Herrera et al., 2012), which similarly employed a checkerboard-based correction paradigm and the same dataset. The experimental results demonstrated comparable correction accuracy between both methods at proximal ranges (<0.3m). However, the proposed algorithm exhibited superior performance at distal ranges (>0.9m). This enhanced long-range accuracy was attributed to the iterative optimization framework, which provided improved initial parameter estimation through successive approximation, thereby reducing error propagation in depth correction.

### 3.3 Scrubbing experiment

The experiment was conducted to quantitatively evaluate the system’s performance during simulated and executed scrubbing tasks. It focused on the system’s ability to ensure the scrubbing head maintained the correct trajectory while moving along the target area.

**FIGURE 10:**
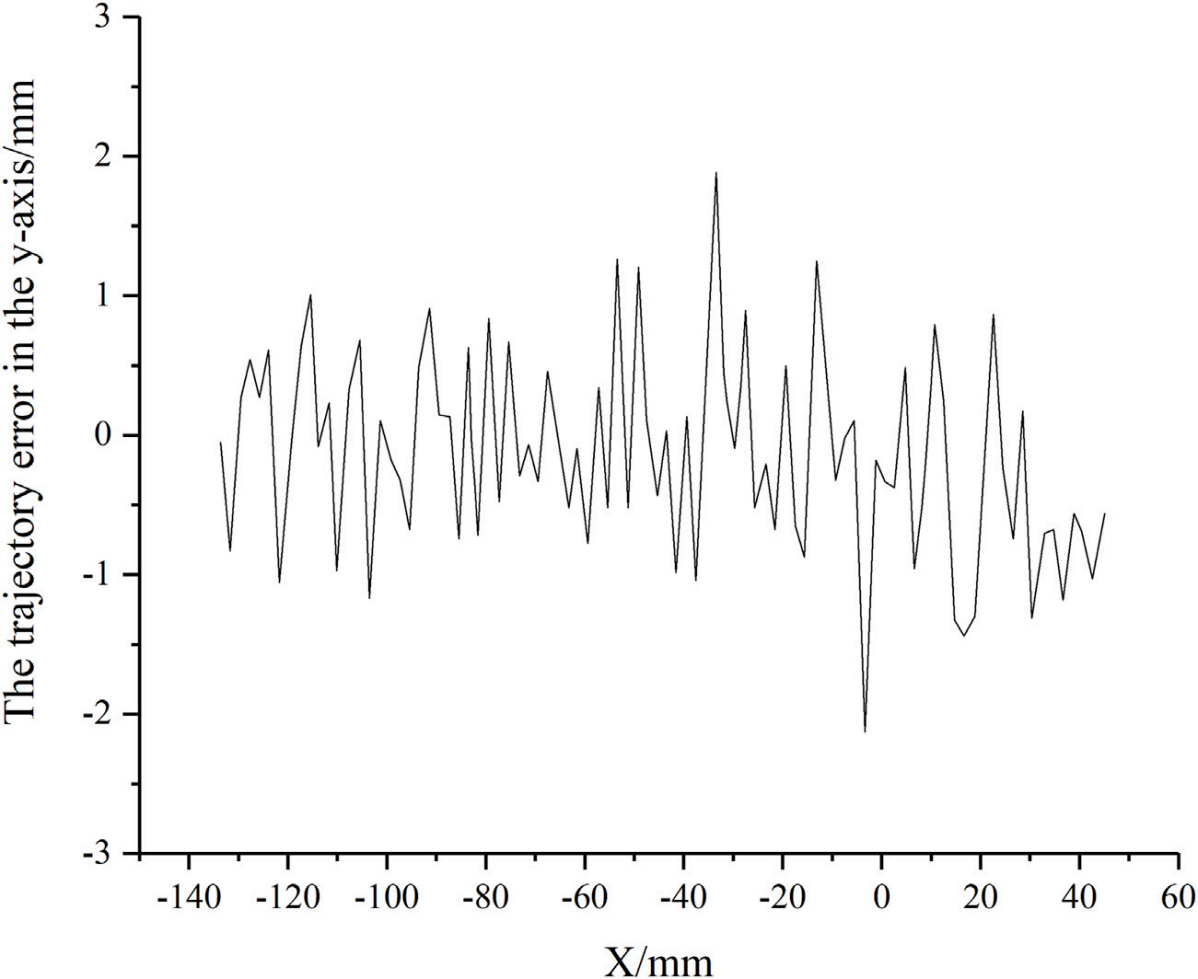
The trajectory error in the *y*-axis.

The scrubbing trajectory was sampled along the end-effector’s x-axis with a point spacing of 2 mm to complete the adaptive scrubbing task. By comparing the actual movement of the scrubbing head with the desired trajectory, the trajectory error in the y-axis was obtained, as shown in Figure 10. The maximum error was 2.23 mm. Given that the adjustable length of the scrubbing head’s brush was 10 mm, the error did not affect the fitting of the scrubbing head to the human skin surface.

## 4 Discussion

Visual perception was frequently employed in robotic perception systems; however, the involvement of skin detection could raise privacy concerns among users. To address these concerns, the following privacy protection strategies were implemented based on practical requirements: 1) A color image encryption scheme based on vector representation was adopted; 2) Noise, such as Gaussian noise and salt-and-pepper noise, was added to the image to obfuscate private information; 3) Data processing occurred exclusively on the local embedded platform, ensuring that no data was transmitted to the cloud for processing; 4) During operation, the visual scenes captured by the sensor were not displayed, and only topics published via ROS were output; 5) Due to the limited storage capacity of the embedded platform, visual data in the bathing scenario was not stored; 6) The system was not connected to the internet, eliminating data transmission risks.

In this study, the six-dimensional force sensor played a crucial role in ensuring safety during emergencies. When the detected force exceeded a predefined threshold, the robot retracted the end-effector and returned to its initial pose. Achieving precise force control typically required more complex control algorithms, particularly when adjustments to all three forces and moments were necessary. However, by utilizing the high-precision distance information fromdepth correction in this study, combined with the safety guarantees provided by the force sensor, the adaptive scrubbing task could be completed without relying on complex control algorithms. The innovation of this work lay primarily in developing an algorithm for enhancing depth quality, which demonstrated excellent performance in both localization and scrubbing. Additionally, the study contributed by creating skin detection datasets, providing valuable data support for related research.

The biological characteristics of the human model differed from the skin properties of real human subjects. Future research will involve experiments on real human participants under ethically approved conditions. Additionally, the current system employed a seated posture with a collaborative robot for bathing tasks, which did not allow effective cleaning of the buttocks region. In future developments, a stand-to-sit posture transition mechanism could be designed to enable buttocks cleaning in a standing position. Furthermore, a user intent recognition module could be incorporated to fully account for the preferences of users, thereby enhancing the system’s intelligence and human-robot interaction experience.

The proposed system demonstrated significant advancements over conventional bathing systems through three key innovations. First, it incorporated a visual perception module to enhance operational intelligence. Second, compared with existing vision-enabled systems, it achieved superior depth measurement accuracy and utilized precise depth information for reliable positioning and scrubbing. Third, the implementation of a motorized rotating seat mechanism reduced the number of required vision sensors while ensuring complete body coverage.

## 5 Conclusion

Acquiring depth information was a critical step for enabling accurate robotic target localization. The intelligent bathing assistance system employed an RGB-D camera to collect depth data and utilized deep learning techniques to detect different skin regions in RGB images, enabling an intelligent selection of bathing modes. The system demonstrated robustness against variations in skin tone, lighting, and humidity. This study proposed a depth correction algorithm to achieve high-precision target localization. During contact, the system did not rely on constant-force control; instead, it leveraged high-accuracy visual data and an adjustable-length scrubbing head to implement strategies including scrubbing trajectory optimization, normal vector estimation, segmented interpolation for real-time planning, and end-effector pose updates. The system’s high-precision localization and adaptive scrubbing capabilities were primarily attributed to the precise visual information obtained through depth correction, eliminating the need for complex force control algorithms.

## Data Availability

The datasets for this study can be found in https://doi.org/10.6084/m9.figshare.21282396.v1 [FigShare].
